# Supplementary material for: Effects of pathogen reproduction system on the evolutionary and epidemiological control provided by deployment strategies for two major resistance genes in agricultural landscapes
Source: Evol Appl. 2023 Dec 19;17(1):e13627. doi: 10.1111/eva.13627 (PMC10810173; doi:10.1111/eva.13627)
Supplement: Supplementary file 1 — Appendix S1. [file EVA-17-e13627-s001.pdf]

# Supporting Information

# Figure S1

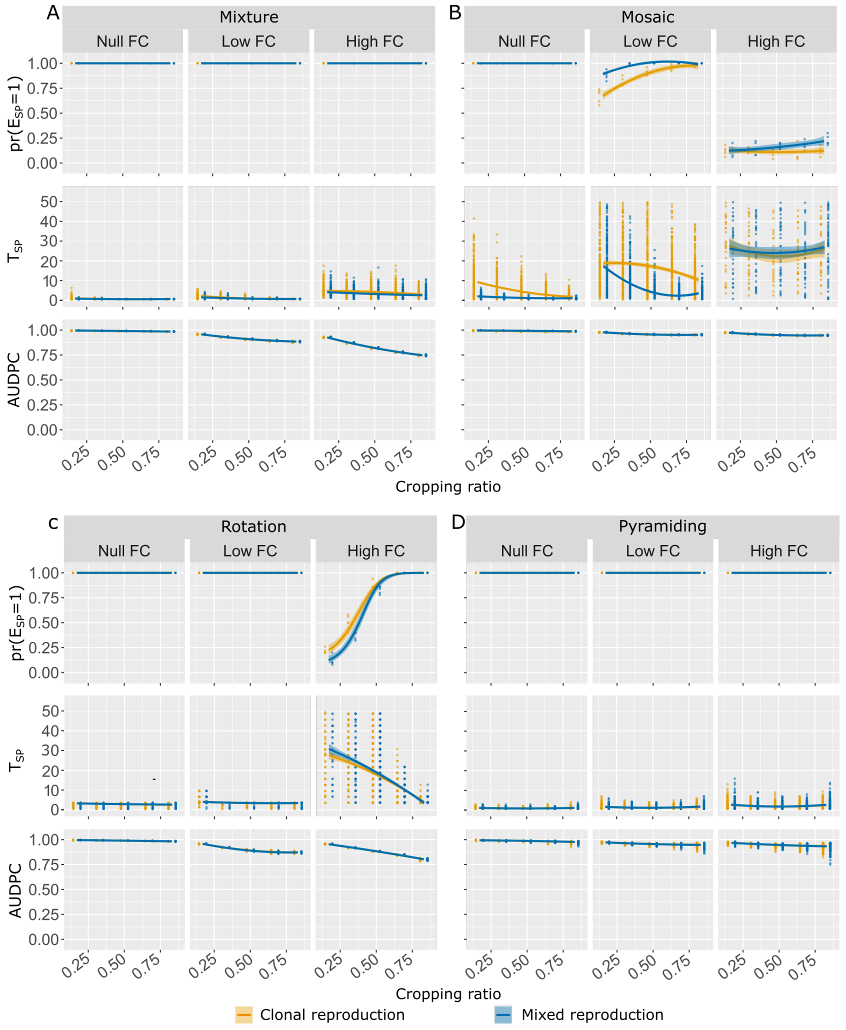


Figure S1: Probability of SP establishment (first row of each panel), time to SP establishment, given the SP gets established, (second row) and AUDPC(third row) at high ($\tau = 10^{-4}$) mutation probability and at null ($\theta = 0$), low ($\theta = 0.25$) and high ($\theta = 0.5$) fitness cost (FC). Panels show the probability of $E_{SP}$, $T_{SP}$, and AUDPC as a function of the cropping ratio for the two pathogen reproduction systems and the four deployment strategies considered. Curved are based on logistic or second order polynomial regression fitting performed on simulations outputs (represented by points, note that in the first row of each panel the points represent the proportion of $E_{SP} = 1$ among the 50 replicas), shaded envelopes delimit the 2.5th and 97.5th percentiles.

## Figure S2

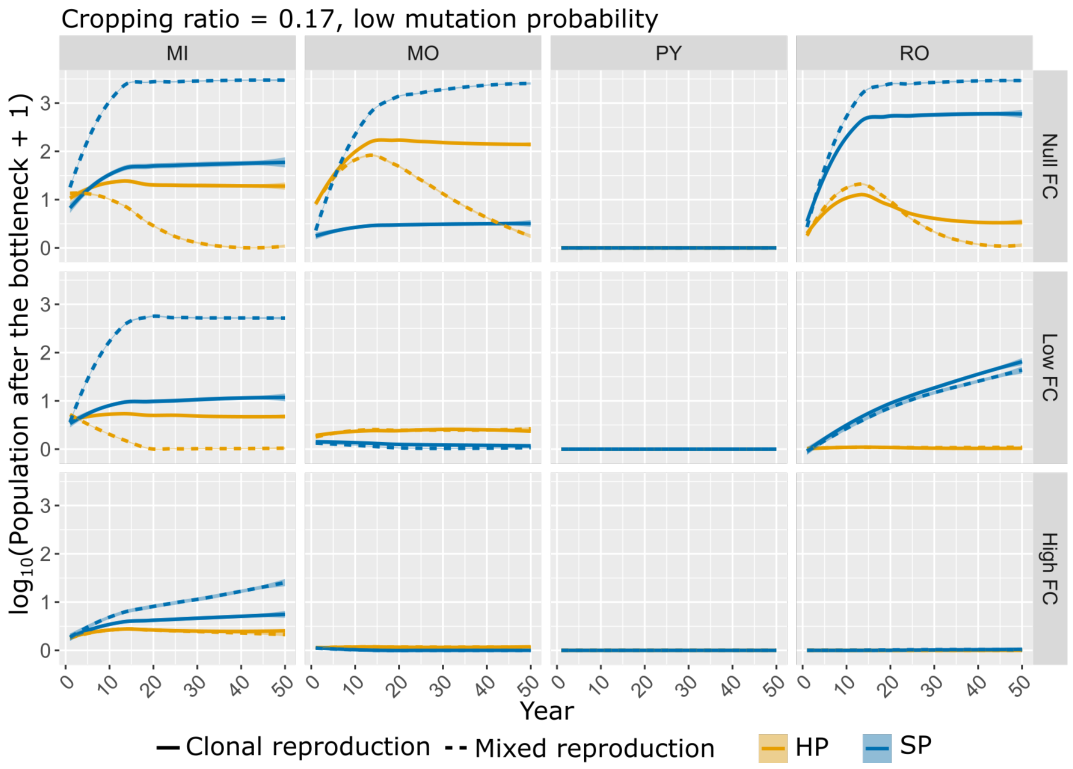


Figure S2: Population size of the superpathogen $SP_{tf}$ (in blue) and maximum number of heterogeneous parental pairs $HP_{tf}$ (in orange) in the landscape after the bottleneck. Curves represent populations dynamics across resistance deployment strategies (MIxture, MOsaic, ROtation and PYramiding) fitness costs and reproduction systems, at low mutation probability ($\tau = 10^{-7}$) and cropping ratio $\varphi = 0.17$. Curves are based on the local polynomial regression fitting performed on simulations outputs. Shaded envelopes delimit the 2.5th and 97.5th percentiles.

## Figure S3

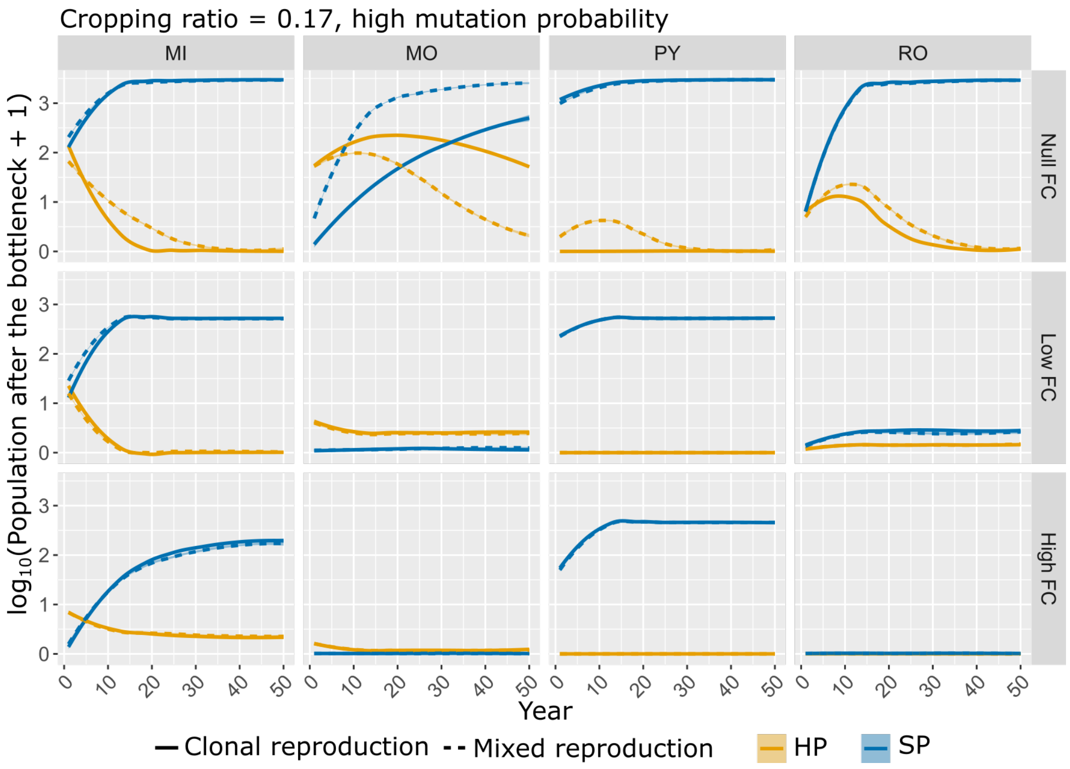


Figure S3: Population size of the superpathogen $SP_{tf}$ (in blue) and maximum number of heterogeneous parental pairs $HP_{tf}$ (in orange) in the landscape after the bottleneck. Curves represent populations dynamics across resistance deployment strategies (MIxture, MOsaic, ROtation and PYramiding) fitness costs and reproduction systems, at high mutation probability ($\tau = 10^{-4}$) and cropping ratio $\varphi = 0.17$. Curves are based on the local polynomial regression fitting performed on simulations outputs. Shaded envelopes delimit the 2.5th and 97.5th percentiles.

## Figure S4

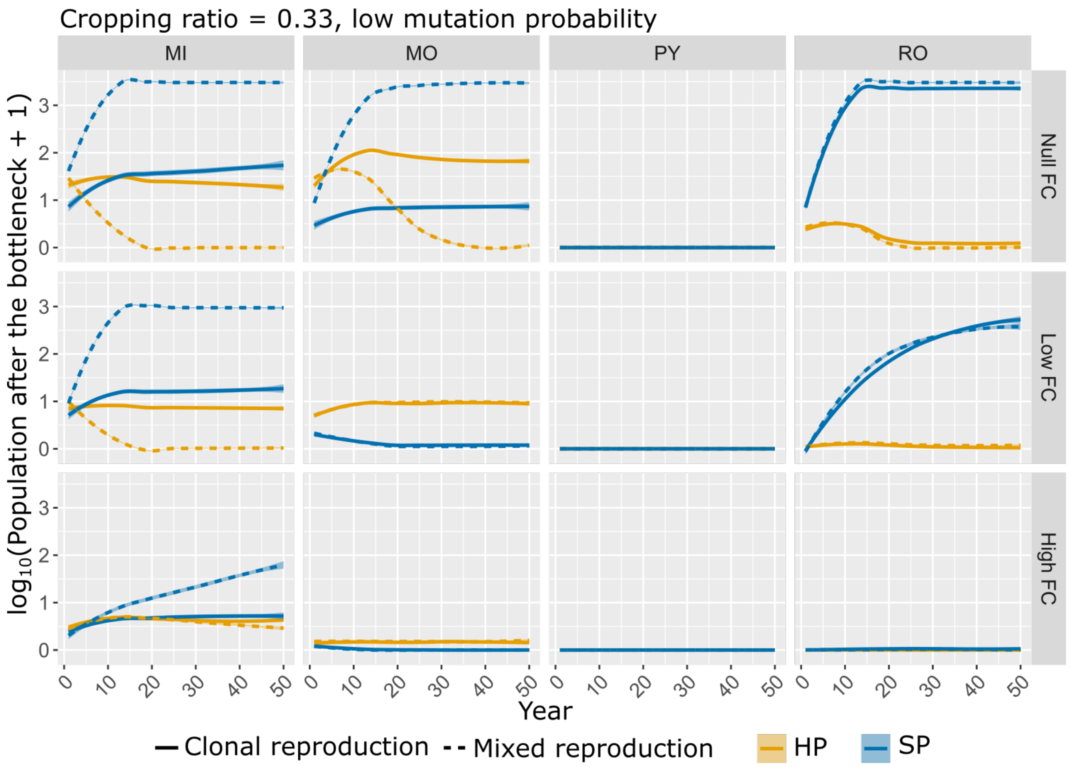


Figure S4: Population size of the superpathogen $SP_{tf}$ (in blue) and maximum number of heterogeneous parental pairs $HP_{tf}$ (in orange) in the landscape after the bottleneck. Curves represent populations dynamics across resistance deployment strategies (MIxture, MOsaic, ROtation and PYramiding) fitness costs and reproduction systems, at low mutation probability ($\tau = 10^{-7}$) and cropping ratio $\varphi = 0.33$. Curves are based on the local polynomial regression fitting performed on simulations outputs. Shaded envelopes delimit the 2.5th and 97.5th percentiles.

## Figure S5

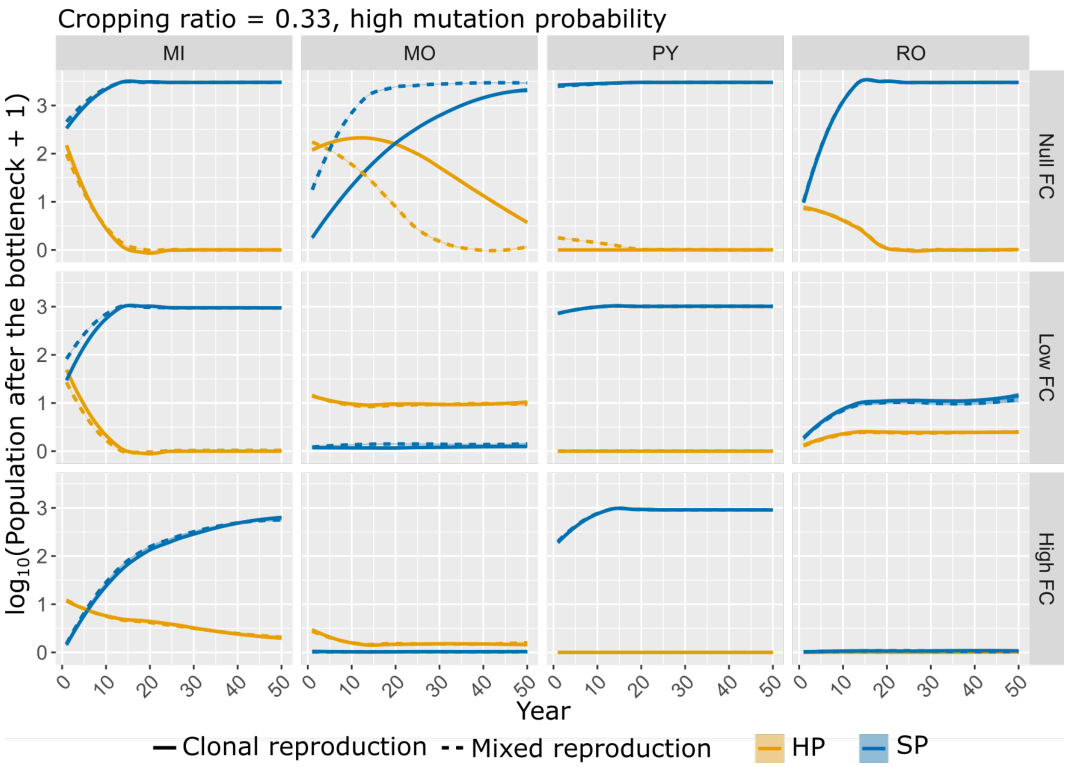


Figure S5: Population size of the superpathogen $SP_{tf}$ (in blue) and maximum number of heterogeneous parental pairs $HP_{tf}$ (in orange) in the landscape after the bottleneck. Curves represent populations dynamics across resistance deployment strategies (MIxture, MOsaic, ROtation and PYramiding) fitness costs and reproduction systems, at high mutation probability ($\tau = 10^{-4}$) and cropping ratio $\varphi = 0.33$. Curves are based on the local polynomial regression fitting performed on simulations outputs. Shaded envelopes delimit the 2.5th and 97.5th percentiles.

## Figure S6

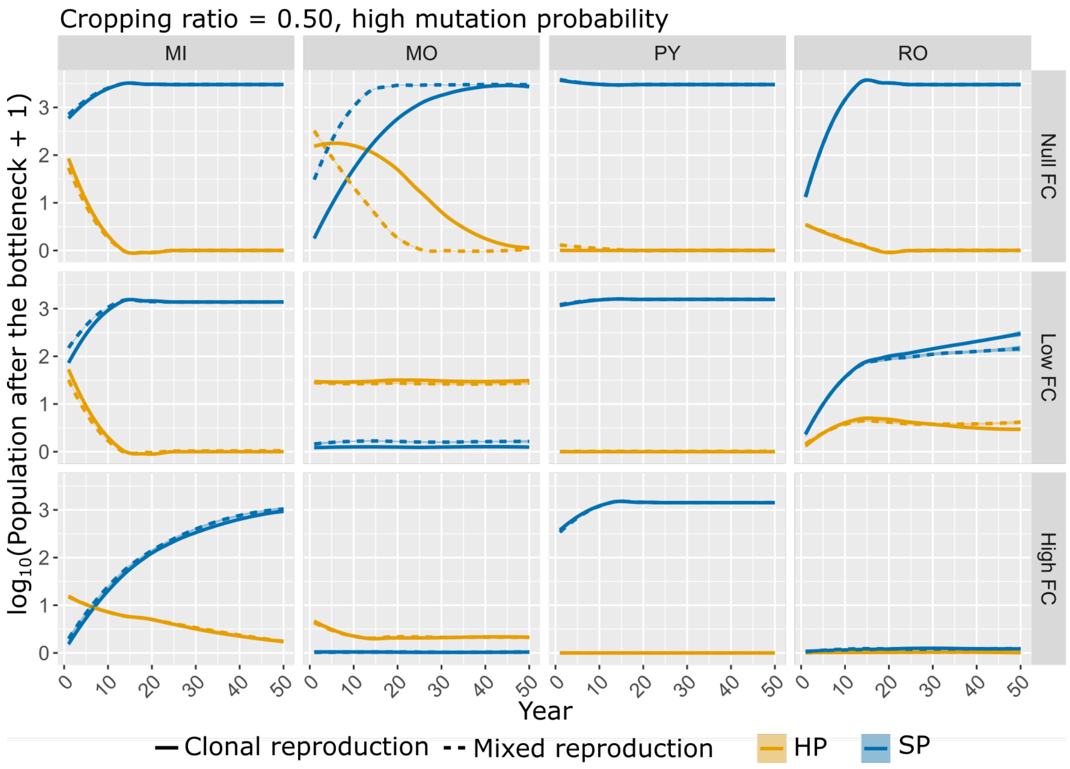


Figure S6: Population size of the superpathogen $SP_{tf}$ (in blue) and maximum number of heterogeneous parental pairs $HP_{tf}$ (in orange) in the landscape after the bottleneck. Curves represent populations dynamics across resistance deployment strategies (MIxture, MOsaic, ROtation and PYramiding) fitness costs and reproduction systems, at high mutation probability ($\tau = 10^{-4}$) and cropping ratio $\varphi = 0.50$. Curves are based on the local polynomial regression fitting performed on simulations outputs. Shaded envelopes delimit the 2.5th and 97.5th percentiles.

## Figure S7

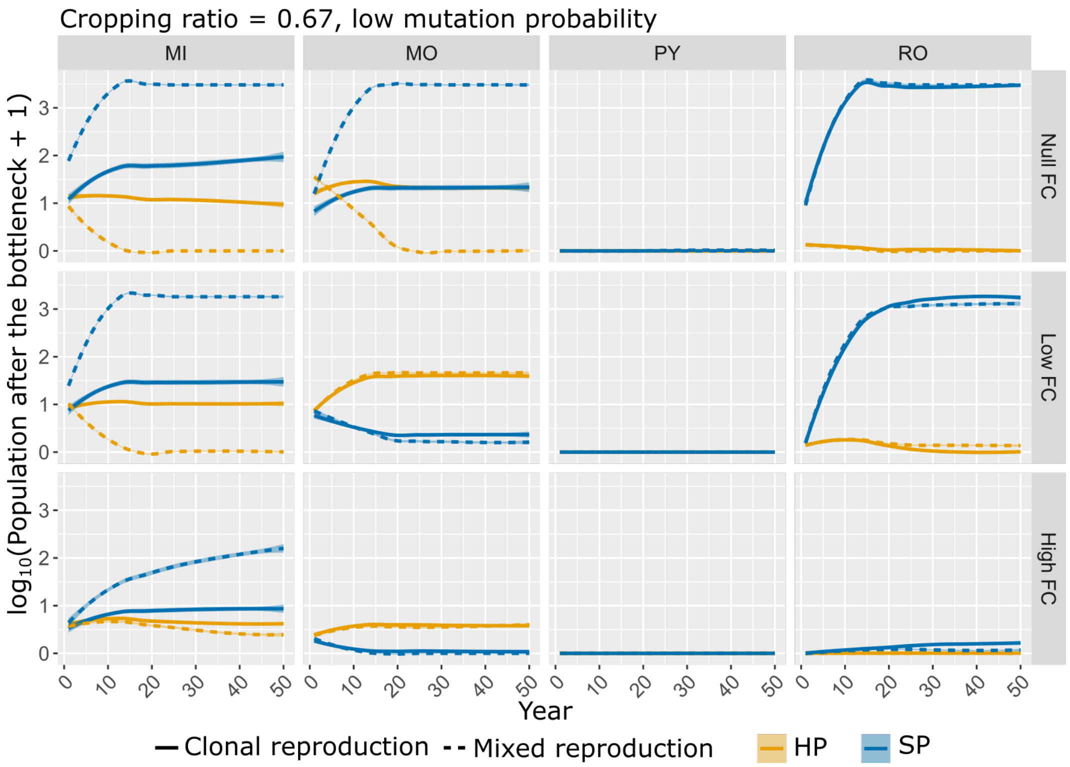


Figure S7: Population size of the superpathogen $SP_{tf}$ (in blue) and maximum number of heterogeneous parental pairs $HP_{tf}$ (in orange) in the landscape after the bottleneck. Curves represent populations dynamics across resistance deployment strategies (MIxture, MOsaic, ROtation and PYramiding) fitness costs and reproduction systems, at low mutation probability ($\tau = 10^{-7}$) and cropping ratio $\varphi = 0.67$. Curves are based on the local polynomial regression fitting performed on simulations outputs. Shaded envelopes delimit the 2.5th and 97.5th percentiles.

## Figure S8

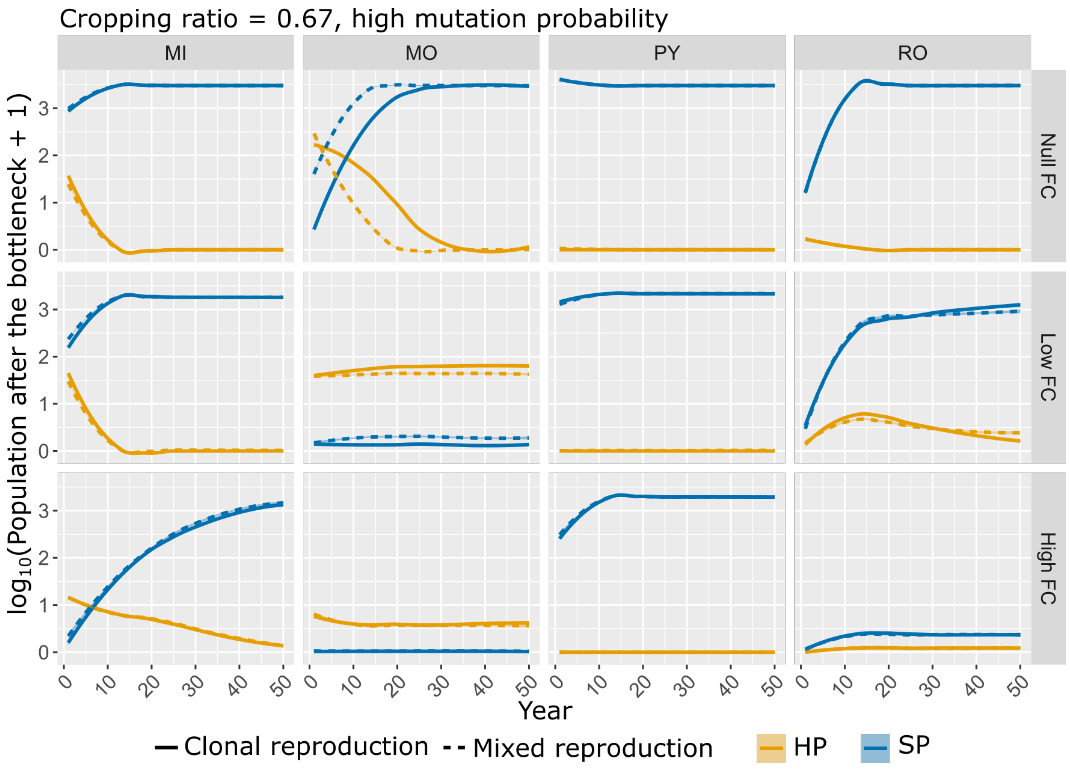


Figure S8: Population size of the superpathogen $SP_{tf}$ (in blue) and maximum number of heterogeneous parental pairs $HP_{tf}$ (in orange) in the landscape after the bottleneck. Curves represent populations dynamics across resistance deployment strategies (MIxture, MOsaic, ROtation and PYramiding) fitness costs and reproduction systems, at high mutation probability ($\tau = 10^{-4}$) and cropping ratio $\varphi = 0.67$. Curves are based on the local polynomial regression fitting performed on simulations outputs. Shaded envelopes delimit the 2.5th and 97.5th percentiles.

## Figure S9

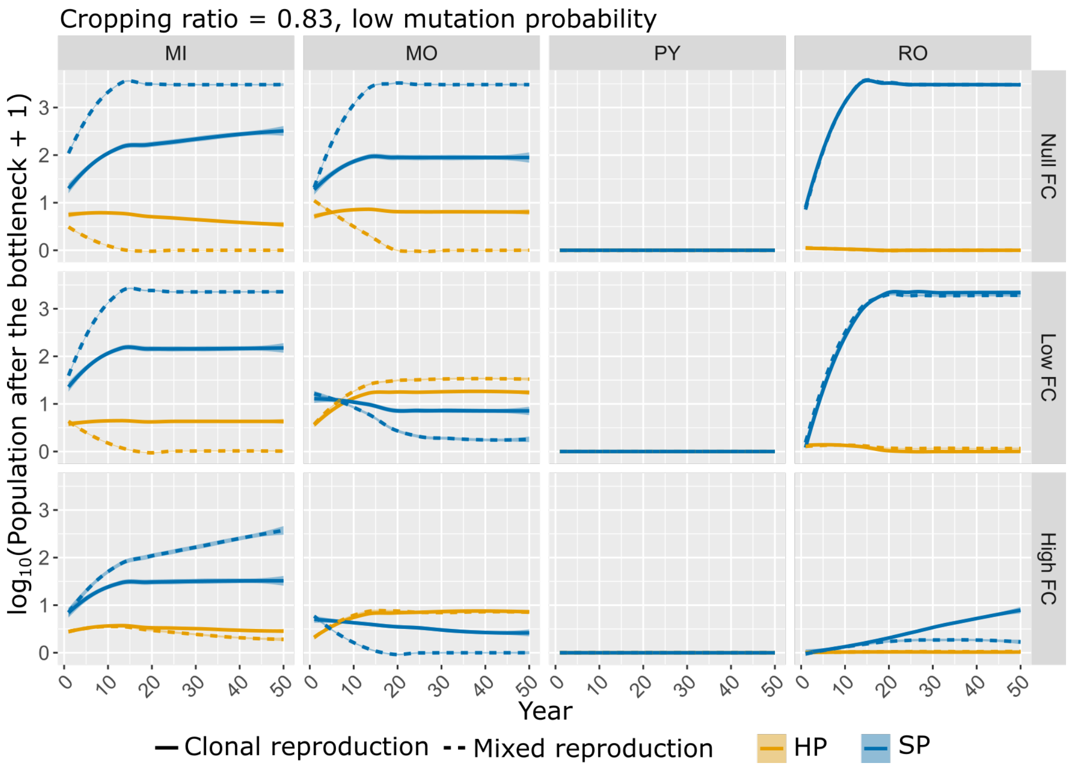


Figure S9: Population size of the superpathogen $SP_{tf}$ (in blue) and maximum number of heterogeneous parental pairs $HP_{tf}$ (in orange) in the landscape after the bottleneck. Curves represent populations dynamics across resistance deployment strategies (MIxture, MOsaic, ROtation and PYramiding) fitness costs and reproduction systems, at low mutation probability ($\tau = 10^{-7}$) and cropping ratio $\varphi = 0.83$. Curves are based on the local polynomial regression fitting performed on simulations outputs. Shaded envelopes delimit the 2.5th and 97.5th percentiles.

## Figure S10

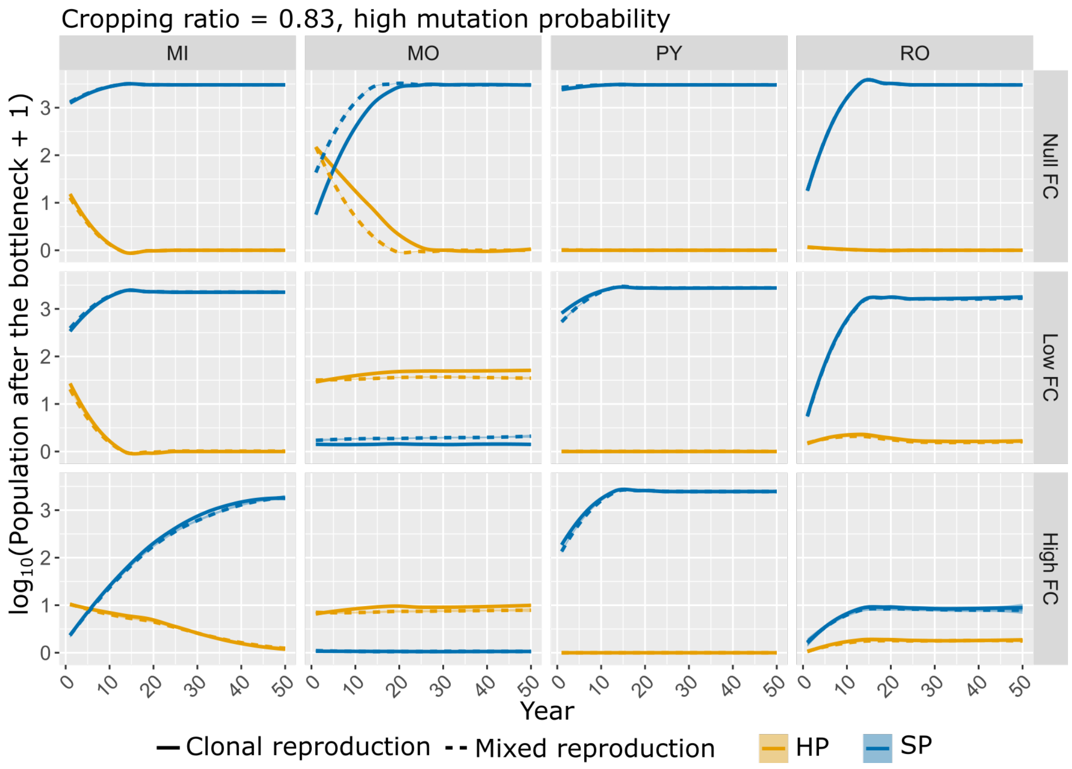


Figure S10: Population size of the superpathogen $SP_{tf}$ (in blue) and maximum number of heterogeneous parental pairs $HP_{tf}$ (in orange) in the landscape after the bottleneck. Curves represent populations dynamics across resistance deployment strategies (MIxture, MOsaic, ROtation and PYramiding) fitness costs and reproduction systems, at high mutation probability ($\tau = 10^{-4}$) and cropping ratio $\varphi = 0.83$. Curves are based on the local polynomial regression fitting performed on simulations outputs. Shaded envelopes delimit the 2.5th and 97.5th percentiles.

**Figure S11**

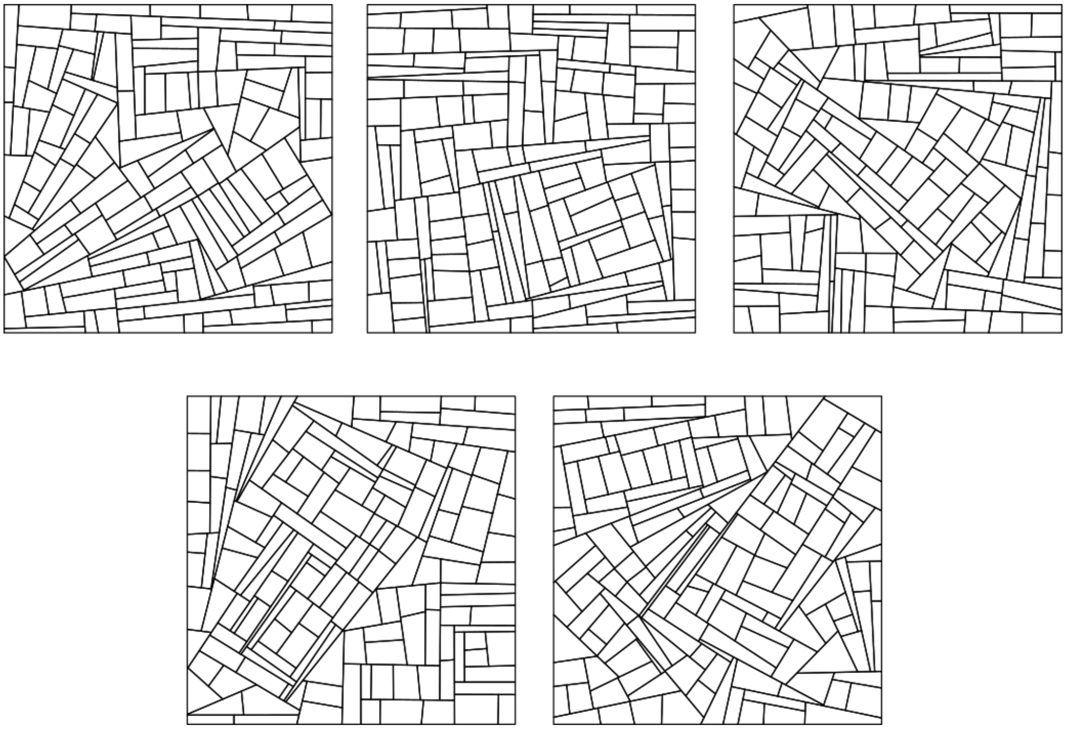

Figure S11: The five landscapes considered in the simulation plan. The landscapes account for 155, 154, 152, 153 and 156 fields, respectively. The total landscape area is 400 ha and the fields area ranges from 0.36 to 5.38 ha (mean: 2.60 ha).

## Note S1 Model assumptions

Here a list of assumptions considered in our model.

1. The spatial unit is a polygon, *i.e.* a piece of land delimited by boundaries and cultivated with a crop. The polygon is considered a homogeneous mixture of host individuals (*i.e.* there is no intra-polygon structuration).

2. Host individuals are in one of these four categories: H (healthy), E (exposed and latent, *i.e.* infected but not infectious nor symptomatic), I (infectious and symptomatic), or R (removed, *i.e.* epidemiologically inactive).

3. A host 'individual' is an infection unit and corresponds to a given amount of plant tissue (where a local infection may develop, *e.g.* fungal lesion). Plant growth increases the amount of available plant tissue (hence the number of individuals) during the cropping season. Plant growth is deterministic (logistic growth) and only healthy individuals (state H) contribute to plant growth (castrating pathogen).

4. The decreasing availability of healthy host tissues (as epidemics spread) makes pathogen infection less likely (*i.e.* density-dependence due to plant architecture).

5. Host are cultivated (*i.e.* planted and harvested), thus there is no host reproduction, dispersal and natural death.

6. Environmental and climate conditions are constant, and host individuals of a given genotype are equally susceptible to disease from the first to the last day of every cropping season.

7. Components of a mixture are independent each other (*i.e.* there is neither plant-plant interaction nor competition for space, and harvests are segregated).

8. The pathogen is considered haploid.

9. Initially, the pathogen is not adapted to any source of resistance, and is only present on susceptible hosts (at state I).

10. Pathogen dispersal is isotropic (*i.e.* equally probable in every direction).

11. Boundaries of the landscape are reflective: propagules stay in the system as if it was closed.

12. Pathogen reproduction can be purely clonal, or mixed (alternation of clonal and sexual reproduction).

13. If there is sexual reproduction (or gene recombination), it occurs only between parental infections located in the same polygon and the same host genotype. The propagule production rate of a parental pair is the sum of the propagule production rates of the parents. For a given parental pair, the genotype of each propagule is issued from random loci segregation of parental qualitative resistance genes.

14. At the end of each cropping season, pathogens experience a bottleneck representing the off-season and then propagules are produced (either via clonal or sexual reproduction). Both clonal and sexual propagules are progressively released during the following season (the day of release of each propagule is sampled from a uniform distribution).

15. Pathogenicity genes mutate independently from each other.

16. Pathogen adaptation to a given resistance gene consists in restoring the same aggressiveness component as the one targeted by the resistance gene.

17. If a fitness cost penalises pathogen adaptation to a given resistance gene, this cost is paid on hosts that do not carry this gene, and consists in a reduction in the same aggressiveness component as the one targeted by the resistance gene.

# Note S2 Model equations

In the present work, the model is an adapted version of the one presented in Rimbaud et al. (2018), which simulates the clonal reproduction, spread and evolution of a pathogen in an agricultural landscape over multiple cropping seasons. Mainly, we introduce between-season sexual reproduction, characteristic of pathogens with a mixed reproduction system. We split the modelled cropping season into two distinct time periods: *i*) within cropping season, where multiple clonal reproduction events take place, and *ii*) between cropping seasons when a single sexual reproduction event may take place. The entire model is described in the following sections. Note that in the equations below only major resistance genes are considered. See Rimbaud et al. (2018) for details on model equations considering both major genes and quantitative resistance traits.

## S2. 1 Host and pathogen demo-genetic dynamics within cropping season

The demo-genetic dynamics of the host-pathogen interaction are based on a HLIR structure ("healthy-latent-infectious-removed"). Thus, in the following, $H_{i,v,t}$, $L_{i,v,p,t}$, $I_{i,v,p,t}$, $R_{i,v,p,t}$, and $Pr_{i,p,t}$ respectively denote the number of healthy, latent, infectious and removed individuals (in this model, an "individual" is a given amount of plant tissue, and is referred to as a "host" hereafter for simplicity), and pathogen propagules in field $i$ ($i$=1,...,$J$), for cultivar $v$ ($v$=1,...,$V$), pathogen genotype $p$ ($p$=1,...,$P$) at time step $t$ ($t$=1,...,$T \times Y$). $T$ is the number of time steps in a cropping season and $Y$ the number of simulated years (*i.e.* cropping seasons). Since the host is cultivated, we assume there is no host reproduction, dispersal or natural mortality (leaf senescence near the end of the cropping season is considered as part of host harvest).

**Host growth.** Only healthy hosts (denoted as $H_{i,v,t}$) are assumed to contribute to growth of the crop. Thus, at each step $t$ during a cropping season, the plant cover of cultivar $v$ in field $i$ increases as a logistic function, and the new amount of healthy plant tissue is:

$$H_{i,v,t+1} = H_{i,v,t}\left[1 + \delta_v \times \left(1 - \frac{N_{i,v,t}}{K_{i,v}}\right)\right] \tag{1}$$

with $\delta_v$ the growth rate of cultivar $v$; $N_{i,v,t} = H_{i,v,t} + \sum_{p=1}^{P}\left(L_{i,v,p,t} + I_{i,v,p,t} + R_{i,v,p,t}\right)$ the total number of hosts in field $i$ for cultivar $v$ and at time $t$; and $K_{i,v} = A_i \times C_v^{max}$ the carrying capacity of cultivar $v$ in field $i$, which depends on $A_i$, the area of the field, and $C_v^{max}$, the maximal density for cultivar $v$. Note that equation (1) adequately approximates a continuous time logistic function only when $\delta_v \leq 1$, otherwise negative recruitment could be generated. When a mixture of several cultivars is present in the same field, decreased growth due to susceptible plants being diseased is not compensated by increased growth of resistant plants.

**Contamination of healthy hosts.** The healthy compartment ($H$) is composed of hosts which are free of pathogen propagules ($H^1$), as well as hosts contaminated (but not yet infected) by the arrival of such propagules ($H^2$). At the beginning of each step, all healthy hosts are considered free of propagules ($H^1$). Then at time $t$ in field $i$ and for cultivar $v$, the number of contaminable hosts (*i.e.* accessible to pathogen propagules, denoted as $H_{i,v,t}^{contaminable}$) depends on the proportion of healthy hosts ($H_{i,v,t}^1$) in the host population ($N_{i,v,t}$):

$$H_{i,v,t}^{contaminable} \sim Binomial\left(H_{i,v,t}^1; \pi\left(\frac{H_{i,v,t}^1}{N_{i,v,t}}\right)\right) \tag{2}$$

with $\pi(x) = \frac{1-e^{-\kappa x^\sigma}}{1-e^{-\kappa}}$, a sigmoid function with $\pi(0) = 0$ and $\pi(1) = 1$, giving the probability for a healthy host to be contaminated. Here, we assume that healthy hosts are not equally likely to be contacted by propagules, for instance because of plant architecture. Moreover, as the local severity of disease increases, eventually the probability for a single propagule to contaminate a healthy host declines due to the decreased availability of host tissue.

Following the arrival of propagules of pathogen genotype $p$ in field $i$ at time $t$ (denoted as $Pr_{i,p,t}^4$, see below), susceptible hosts become contaminated. The pathogen genotypes of these propagules are distributed among contaminable hosts according to their proportional representation in the total pool of propagules. Thus, for cultivar $v$, the vector describing the maximum number of contaminated hosts by each pathogen genotype (denoted as $[H_{i,v,t}^{maxConta}]_{p=1,...,P}$) is given by a multinomial draw:

$$[H_{i,v,t}^{maxConta}]_{p=1,...,P} \sim Multinomial\left(H_{i,v,t}^{contaminable}; \left[\frac{Pr_{i,p,t}^4}{\sum_{p=1}^{P} Pr_{i,p,t}^4}\right]_{p=1,...,P}\right) \tag{3}$$

However, the number of deposited propagules ($Pr_{i,p,t}^4$) may be smaller than the maximal number of contaminated hosts ($H_{i,v,p,t}^{maxConta}$). Thus, the true number of hosts of cultivar $v$, contaminated by pathogen genotype $p$ in field $i$ at $t$ (denoted as $H_{i,v,p,t}^2$) is given by:

$$[H_{i,v,p,t}^1 \rightarrow H_{i,v,p,t}^2] = min\left(H_{i,v,p,t}^{maxConta}; Pr_{i,p,t}^4\right) \tag{4}$$

**Infection.** Between $t$ and $t+1$, in field $i$, contaminated hosts ($H_{i,v,p,t}^2$) become infected (state L) with probability $e_{v,p}$, which depends on the maximum expected infection probability, $e_{max}$, and the interaction between host ($v$) and pathogen ($p$) genotypes:

$$[H_{i,v,p,t}^2 \rightarrow L_{i,v,p,t+1}] \sim Binomial\left(H_{i,v,p,t}^2; e_{v,p}\right) \tag{5}$$

$$e_{v,p} = e_{max} \times \prod_{g=1}^{G} INF_{ig_g(p),mg(v)}^g \tag{6}$$

$INF^g$ represent the infectivity matrix for major gene $g$ which summarizes the possible interactions between potential host resistance genes ($mg$) and associated pathogen infectivity genes ($ig$), see Table 1 in the main text for an example.

**Latent period.** Infected hosts become infectious (state I) after a latent period (LI) drawn from a Gamma distribution (a flexible continuous distribution from which durations in the interval $[0; +\inf[$ can be drawn) parameterised with expected value, $\Gamma_{exp}$, and variance, $\Gamma_{var}$:

$$(LI) \sim Gamma(\Gamma_{exp}; \Gamma_{var}) \tag{7}$$

Note, the usual shape and scale parameters of a Gamma distribution, $\beta_1$ and $\beta_2$, can be calculated from the expectation and variance, exp and var, with: $\beta_1 = \frac{exp^2}{var}$ and $\beta_2 = \frac{var}{exp}$, respectively.

**Infectious period.** Finally, infectious hosts become epidemiologically inactive (*i.e.* they no longer produce propagules, thus are in state R, "removed") after an infectious period (IR) drawn from a Gamma distribution parameterised with expected value, $\Upsilon_{exp}$ and variance, $\Upsilon_{var}$, similar to the latent period:

$$(IR) \sim Gamma(\Upsilon_{exp}; \Upsilon_{var}) \tag{8}$$

**Pathogen clonal reproduction.** In field $i$ at time $t$, infectious hosts associated with pathogen genotype $p$ produce a total number of propagules (denoted as $Pr^1_{i,p,t}$), drawn from a Poisson distribution with parameter $r_{exp}$ corresponding to the expected number of propagules produced by a single infectious host per time step:

$$Pr^1_{i,p,t} \sim Poisson(r_{exp} \sum_{v=1}^{V} I_{i,v,p,t}) \tag{9}$$

**Pathogen mutation.** The following algorithm is repeated independently for every potential infectivity gene g:

1. the pathotype (*i.e.* the level of adaptation with regard to major gene $g$, indexed by $q$; $q = 1, \ldots, Q_g$; with $Q_g = 2$ since the pathotype is either infective, or non-infective) of the pathogen propagules is retrieved from their genotype $p$;
2. propagules can mutate from pathotype $q$ to pathotype $q'$ with probability $m^g_{qq'}$ such as $m^g_{qq'} = \tau_g$ if $q' \neq q$ (hence $m^g_{qq} = 1 - \tau_g$ since $Q_g = 2$). Thus, in field $i$ at time $t$, the vector of the number of propagules of each pathotype arising from pathotype $q$ (denoted as $[M^g_{i,q,q',t}]_{q'=1,\ldots,Q_g}$) is given by a multinomial draw:

$$[M^g_{i,q,q',t}]_{q'=1,\ldots,Q_g} \sim Multinomial\left(Pr^1_{i,p,t}; [m^g_{qq'}]_{q'=1,\ldots,Q_g}\right) \tag{10}$$

3. the total number of propagules belonging to pathotype $q'$ and produced in field $i$ at time $t$ (denoted as $Pr^2_{i,q',t}$) is:

$$Pr^2_{i,q',t} = \sum_{q=1}^{Q_g} M^g_{i,q,q',t} \tag{11}$$

4. the new propagule genotype $p'$ is retrieved from its new pathotype ($q'$), and the number of propagules is incremented using a variable denoted as $Pr^3_{i,p',t}$.

In this model, it should be noted that the mutation probability $\tau_g$ is not the classic mutation rate (*i.e.* the number of genetic mutations per generation per base pair), but the probability for a propagule to change its infectivity on a resistant cultivar carrying major gene $g$. This probability depends on the classic mutation rate, the number and nature of the specific genetic mutations required to overcome major gene $g$, and the potential dependency between these mutations.

**Pathogen dispersal.** Propagules (both clonal and sexual, see below) can migrate from field $i$ (whose area is $A_i$) to field $i'$ (whose area is $A'_i$) with probability $\mu_{ii'}$, computed from:

$$\mu_{ii'} = \frac{\int_{A_i}\int_{A_{i'}} g(\|z' - z\|)dzdz'}{A_i} \tag{12}$$

with $\|z' - z\|$ the Euclidian distance between locations $z$ and $z'$ in fields $i$ and $i'$, respectively, and $g(\|z' - z\|) = \frac{(b-2)(b-1)}{2\pi a^2}\left(1 + \frac{\|z'-z\|}{a}\right)^{-b}$ the two-dimensional power law dispersal kernel of the propagules. The computation of $\mu_{ii'}$ probability is performed using the *CaliFloPP* algorithm (Bouvier et al., 2009). Thus, at time $t$, the vector of the number of propagules of genotype $p$ migrating from field $i$ to each field $i'$ (denoted as $[D_{i,i',p,t}]_{i'=1,...,J}$) is:

$$[D_{i,i',p,t}]_{i'=1,...,J} \sim Multinomial(Pr^3_{i,p,t}; [\mu_{i,i'}]_{i'=1,...,J}) \tag{13}$$

and the total number of propagules arriving in field $i'$ at time $t$ (denoted as $Pr^4_{i',p,t}$) is:

$$Pr^4_{i',p,t} = \sum_{i=1}^{J} D_{i,i',p,t} \tag{14}$$

We consider that propagules landing outside the boundaries of the simulated landscape are lost (absorbing boundary condition), and there are no propagule sources external to the simulated landscape.

## S2. 2 Host and pathogen demo-genetic dynamics between cropping seasons

**Seasonality.** Let $t^0(y)$ and $t^f(y)$ denote the first and last days of cropping season $y$ ($y = 1, \ldots, Y$), respectively. The plant cover in field $i$ for cultivar $v$ at the beginning of cropping season $y$ is set at $H_{i,v,t^0(y)} = A_i \times C_v^0 \times \mathbb{I}_{v(i)=v}$, with $C_v^0$ the plantation density of cultivar $v$ and $\mathbb{I}_{v(i)}$ an indicative variable set at 1 when field $i$ is cultivated with cultivar $v$ and 0 otherwise. At the end of a cropping season, the host is harvested. We assume that the pathogen needs a green bridge to survive the off-season. This green bridge could, for example, be a wild reservoir or volunteer plants remaining in the field (*e.g.* owing to incomplete harvest or seedlings). The size of this reservoir imposes a bottleneck for the pathogen population. The number of remaining infected hosts in field $i$ for cultivar $v$ and pathogen genotype $p$ (denoted by $I^*_{i,v,p,t^f(y)}$) at the end of the off-season is given by:

$$I^*_{i,v,p,t^f(y)} \sim Binomial(L_{i,v,p,t^f(y)} + I_{i,v,p,t^f(y)}; \lambda) \tag{15}$$

with $\lambda$ the survival probability of infected hosts. Considering that those hosts produce propagules during their whole infectious period, we compute an equivalent number of infectious hosts by multiplying $I^{eq}_{i,v,p,t^f(y)} = \Upsilon_{exp} \times I^*_{i,v,p,t^f(y)}$. The remaining hosts $I^{eq}_{i,v,p,t^f(y)}$ produce clonal and sexual propagules. Clonal propagules can mutate exactly as happens during the cropping season. The production of propagules through sexual reproduction and the possible genetic recombination are detailed in the following section. Propagules, either clonal or sexual, produced between cropping seasons are uniformly released throughout the following cropping season, constituting the primary inoculum.

**Pathogen sexual reproduction.** In field $i$, the pool of infectious hosts associated to the same cultivar $v$, $I_{i,v,p,t^f(y)}$, undertakes sexual reproduction. Two parental infectious hosts, respectively infected by pathogens $Par_1$ and $Par_2$ are randomly sampled without replacement from the pool of infectious hosts. The couple $c = \{Par_1; Par_2\}$ produce $P^{sex}_{v,c}$ propagules, drawn from a Poisson distribution whose expectation is the sum of the number $r_{exp}$ of propagules produced by each of the parental infectious hosts:

$$P^{sex}_{v,c} \sim Poisson(r_{expv,c} = r_{exp} + r_{exp} = 2 \times r_{exp}) \tag{16}$$

Then the genotype of each propagule is retrieved from parental genotypes: the genotype at every locus $g$ is randomly sampled between one of the two parents $\{Par_1; Par_2\}$. For example, assuming that parental infection $Par_1$ carries infectivity genes to resistance gene $R_1$ (which corresponds to a genotype "10") and parental infection $Par_2$ carries the infectivity genes to resistance $R_2$ (genotype "01"), the resulting propagule genotype could be either the same as one of the two parents, or a superpathogen genotype "11", or a wild-type genotype "00". This process is iterated for all the pairs $c$ = 1,..., $C$ of infectious hosts

associated to all the cultivars $v = 1,...,\ V$ in a given field $i$, resulting in a total number of sexual propagules:

$$P_i^{sex} = \sum_{v=1}^{V} \sum_{c=1}^{C} P_{v,c}^{sex} \tag{17}$$

## S2. 3 Initial conditions

At the beginning of a simulation, healthy hosts are planted in each field. The initial pathogen population is assumed to be totally non-adapted to the resistance genes, and is only present in susceptible fields with probability $\Phi$. Then the initial number of infectious hosts in these fields is:

$$I_{i,v=1,p=1,t=1} \sim Binomial(H_{i,v=1,t=1}; \Phi) \tag{18}$$

# Note S3 Model parameterisation to downy mildew

This section details the parameterisation of the model to approximate the causal agent of downy mildew disease of grapewine, caused by the oomycete *Plasmopara viticola.*

## S3. 1 Pathogen dispersal

Based on the results of previous empirical and modelling studies (Ojiambo et al., 2017; Mundt et al., 2009), the power-law has a good ability to predict the dispersal of aerially transmitted pathogens, assuming it is isotropic (*i.e.* uniform in all directions). We consequently use this function in our model:

$$g(\|z' - z\|) = \frac{(b-2)(b-1)}{2 \cdot \pi \cdot a^2} \left(1 + \frac{\|z' - z\|}{a}\right)^{-b} \tag{19}$$

where $a > 0$ is a scale parameter and $b > 2$ determines the weight of the dispersal tail. The expected dispersal distance is given by: $\mu_{exp} = \frac{2a}{(b-3)}$.

To our knowledge, there are no studies so far estimating the dispersal kernel, or at least an average dispersal distance, for *P. viticola* at a regional scale. Therefore, following previous works estimating the dispersal kernels of airborne plant fungi, we set the mean dispersal distance of a *P. viticola* spore at $\mu_{exp}$= 20 m, which represents 1% of landscape length (Rimbaud et al., 2018) and $b = 3.5$ (Frantzen and Van den Bosch, 2000; Grosdidier et al., 2018). Consequently, the value $a$ was estimated as $a = (b-3) \times \mu_{exp}/2 = 5$.

## S3. 2 Infection probability

In an experiment on the influence of environmental conditions on sporulation of *Plasmopara viticola* lesions, the probability that the inoculum drops deposited on a leaf triggers an infection was estimated to lie between 0.10 and 0.90, depending on the temperature and humidity (Caffi et al., 2016). In the present study, given that the propensity of a leaf to be infected is explicitly modelled by a sigmoid contamination function, the maximal expected infection probability was set at $e_{max} = 0.90$.

## S3. 3 Sporulation rate

The real number of spores produced daily by a lesion and effectively dispersed to a leaf where they may trigger an infection, often summarised by 'effective sporulation rate' and denoted as $r_{exp}$ here, is extremely complicated to estimate. As an indication, the basic reproductive number for a pathogen (*i.e.* the theoretical number of secondary infections from a single infectious host (Anderson and May, 1992)) would be $R_{0max} = e_{max} \times \Upsilon_{exp} \times \mathrm{r}_{exp}$. The $R_0$ of *P. viticola* have been estimated to vary between 0.1-50 (Rossi et al., 2009). Thus, in order to simulate a reasonably aggressive pathogen, and since very few data

were available to quantify $r_{exp}$ compared to other parameters, $r_{exp}$ has been adjusted to be 2 spores.day$^{-1}$, thus $R_{0max}$ = 25.05 (mean value of the range proposed by Rossi et al., 2009).

## S3. 4 Duration of the latent and sporulation periods

We found 13 estimates from 12 studies of *P. viticola* which included information on the duration of the latent period; similarly, there were 6 estimates from 5 studies with data on the sporulation period. When available, standardised conditions in growth chambers were preferred over field data to facilitate comparison between studies. A Gamma distribution was fit to the data using a maximum likelihood approach (figures S12 and S13). From this analysis, the parameters associated with latent and sporulation periods were estimated as: $\gamma_{min} = 7$; $\gamma_{var} = 8$; $\Upsilon_{exp} = 14$ and $\Upsilon_{var} = 22$.

Table S1: Available data on the duration of latent and sporulation periods for downy mildew caused by *P. viticola* (and *formae speciales*). In the following studies, latent and sporulation periods were determined on fully susceptible hosts under controlled condition (cyan cells), in field studies (green cells) or they are derived by biological evidences or other modelling frameworks (yellow cells).

| Reference | Organism | Disease | Latent Period (days) | | | Sporulation perido (days) | | |
|---|---|---|---|---|---|---|---|---|
| | | | min | max | mean | min | max | mean |
| Delmas et al. (2016) | *P. viticola* | Downey mildew | 2.8 | 3.5 | 3.15 | | | |
| Lalancette et al. (1988) | *P. viticola* | Downey mildew | 7 | 12 | 10 | | | |
| Bove et al. (2020) | *P. viticola* | Downey mildew | 6 | 10 | 8 | 15 | 20 | 17.5 |
| Caffi et al. (2013) | *P. viticola* | Downey mildew | 4 | 23 | 11.3 | 2 | 10 | 6.7 |
| Lalancette (1988) | *P. viticola* | Downey mildew | | | 7 | | | |
| Kennelly et al. (2005) | *P. viticola* | Downey mildew | 7 | 10 | 8.5 | | | |
| Mouafo-Tchinda et al. (2020) | *P. v. riparia* | Downey mildew | 5.75 | 6.25 | 6 | | | |
| Mouafo-Tchinda et al. (2020) | *P. v. aestivalis* | Downey mildew | 3.75 | 4.25 | 4 | | | |
| Rumbolz et al. (2002) | *P. viticola* | Downey mildew | 5 | 6 | 5.5 | | | |
| Boso and Kassemeyer (2008) | *P. viticola* | Downey mildew | | | 5 | | | |
| Rossi et al. (2009) | *P. viticola* | Downey mildew | | | 8 | | | 15 |
| Clark and Spencer-Phillips (2004) | *P. viticola* | Downey mildew | 3 | 24 | 13.5 | | | |
| Angelotti. (2017) | *P. viticola* | Downey mildew | 5 | 9 | 7 | | | |
| Bove et al. (2019) | *P. viticola* | Downey mildew | | | | 6 | 30 | 18 |
| Kennelly et al. (2007) | *P. viticola* | Downey mildew | | | | 6 | 18 | 12 |

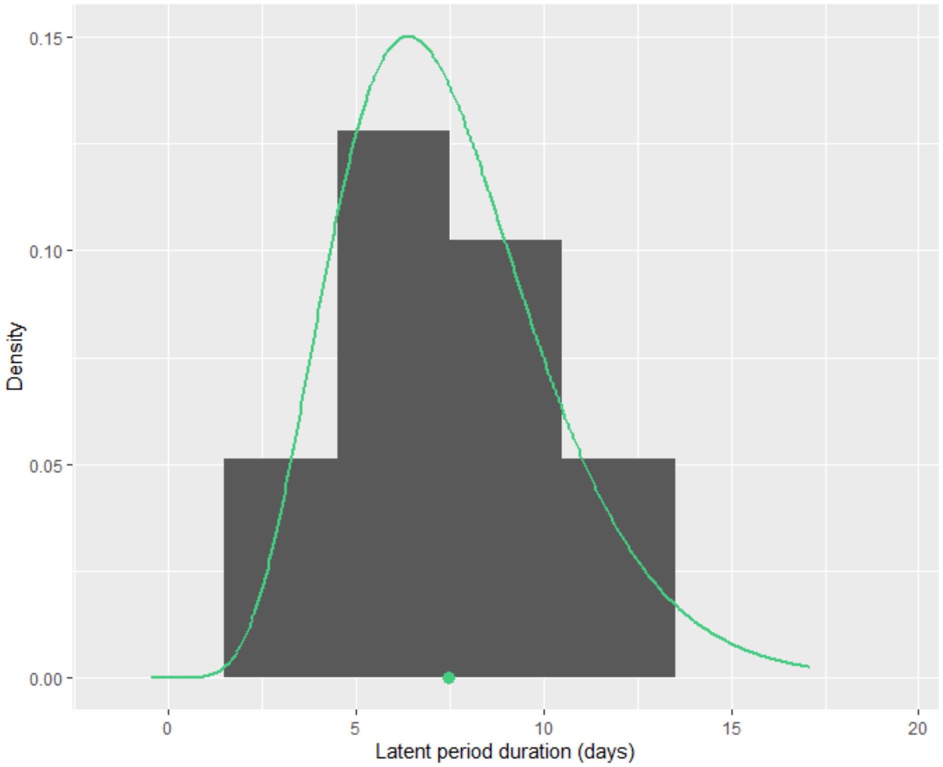


Figure S12: Distribution of the latent period duration of downy mildew caused by *Plasmopora viticola*. Raw data were obtained from previous studies (see Table S1). Green curve is Gamma distribution estimated from the data through maximum likelihood (function fitdistr of the R package MASS, v 7.3-53.1); dot indicate the mean latent period (7.46 days). The variance is 8.02 days.

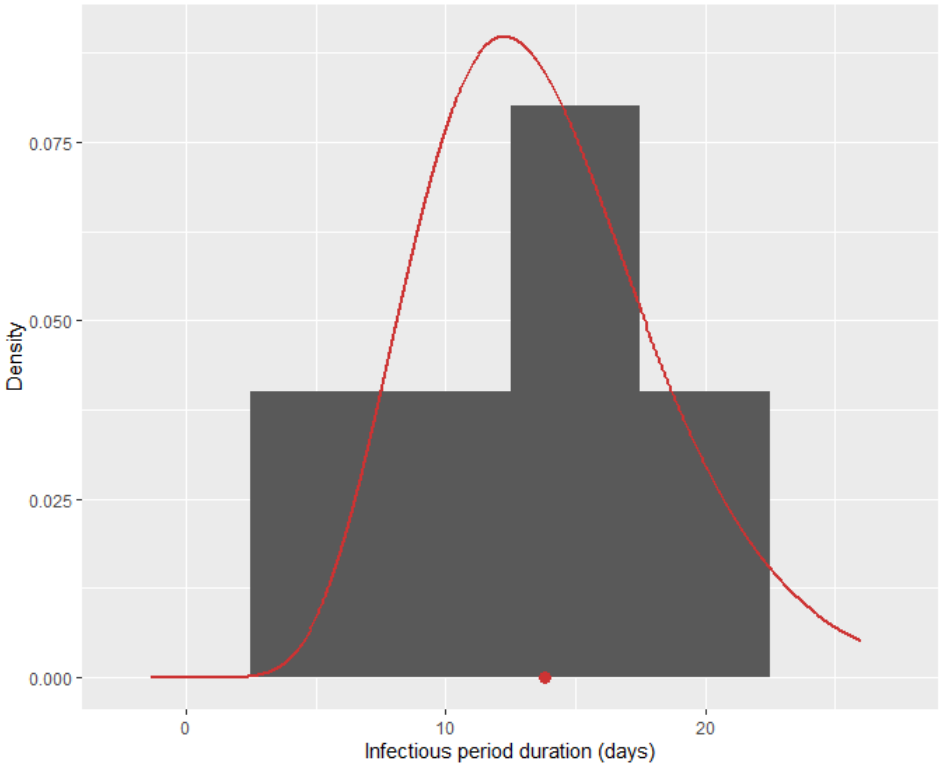


Figure S13: Distribution of the infectious period duration of downy mildew caused by *Plasmopora viticola*. Raw data were obtained from previous studies (see Table S1). Red curve is Gamma distribution estimated from the data through maximum likelihood (function fitdistr of the R package MASS, v 7.3-53.1); dot indicate the mean infectious period (13.84 days). The variance is 21.82 days.

## S3. 5 Contamination function

We assume that susceptible plants become less easily contaminated when the local disease level increases by using the following sigmoid function for $\pi(x)$:

$$\pi(x) = \frac{1 - exp(-\kappa x^{\sigma})}{1 - exp(-\kappa)} \tag{20}$$

The parameters of the sigmoid curve ($\kappa = 5.33, \sigma = 3$) have been parameterised as in previous works (Papaïx et al., 2014b,a, 2015; Rimbaud et al., 2018), such as $\pi(0) = 0, \pi(1) = 1$, and the inflexion point is located at $x_0 = [(\sigma-1)/(\kappa\sigma)]^{1/\sigma} \approx 0.5$. The use of a sigmoid function instead of a linear function implies that the contamination of a susceptible plant is easier when the proportion of susceptible

plants is higher than 50%, and harder when the proportion of susceptible plants is lower than 50%.

# Note S4 Calculation of the threshold for pathogen establishment considering sexual reproduction

In this work, we defined a threshold (denoted by N hereafter) for the number of infections of resistant hosts (considering a constant and infinite host population size) by mutant pathogens with genotype $m_1$, above which they are unlikely to go extinct. The time passed between the beginning of a simulation and the moment the number of infections of resistant hosts by mutant pathogens $m_1$ exceeds the threshold N is considered the time of genotype $m_1$ establishment.

The spread of the mutant genotype $m_1$ across years from two parental infections of a resistant host (for subsequent sexual reproduction) requires surviving the bottleneck imposed by host harvest (denoted by event $Surv_I$), the production of at least one propagule with genotype $m_1$ via sexual reproduction (denoted by the event $Rec_I|Surv_I$) and the infection of new resistant hosts in the next cropping season (denoted by event $Inf_I|Surv_I, Rec_I$). Thus the probability of spread of the mutant genotype $m_1$ across years (event $Inf_I$) is:

$$P(Inf_I) = P(Surv_I) \times P(Rec_I|Surv_I) \times P(Inf_I|Surv_I, Rec_I) \tag{21}$$

The probability for a single infection to survive the bottleneck imposed by host harvest and the off-season corresponds to the off-season survival probability $\lambda$. However, as sexual reproduction takes place, we need, at least, that two infections survive the bottleneck. In a population of N infections, the probability of *i*) none of the infections survives the bottleneck OR *ii*) just one infection survives the bottleneck is :

$$P(Ext) = (1-\lambda)^N + \lambda(1-\lambda)^{N-1} \tag{22}$$

Consequently, the probability that at least two infections survives the bottleneck is:

$$P(Surv_I) = 1 - [(1-\lambda)^N + \lambda(1-\lambda)^{N-1}] \tag{23}$$

We now assume that just two infections survive the bottleneck and sexually reproduce. The probability for a single propagule produced by the couple to have the same genotype $m_1$ of the parental infection $p_1$ is equal to 1 if the other parental infection $p_2$ has genotype $m_1$, too. By contrast if the parental infection $p_2$ has a genotype different from $p_1$, the probability for a single propagule to have genotype $m_1$ is:

$$P(Rec|m_1) = \prod_{g=1}^{G} \frac{1}{ig_g} \tag{24}$$

Where $G$ is the number of resistance genes considered and $ig_g$ is the number of level of aggressiveness for the resistance gene g (=2 for major genes, higher than 2 for QTLs). Note that in equation (4) we are considering the worst case

scenario, that is the two parental infections have different aggressiveness for each resistance gene. If the parental infections $p_1$ and $p_2$ have the same level of aggressiveness for some resistance genes, $P(Rec|m_1)$ would be higher than that in equation (4). In our case, we consider two (G=2) major resistance genes (ig=2), then $P(Rec|m_1) = 0.25$. Consequently, the probability that the produced propagule does not inherit the genotypes $m_1$ from parent $p_1$ is $1 - \prod_{g=1}^{G} \frac{1}{ig_g} = 0.75$. Since two parental infections produces a total of $2 \times r_{exp} \times \Gamma_{exp}$ propagules, the probability that none of the propagules inherit the genotype $m_1$ is $P(Ext_{REC}) = 0.75^{(2 \times r_{exp} \times \Gamma_{exp})}$. Given the values $r_{exp} = 2$, $\Gamma_{exp} = 14$ (parameters for downy mildew), we have $P(Ext_{REC}) \approx 1 \times 10^{-7}$. Consequently, the probability of production of at least one propagule with genotype $m_1$ via sexual reproduction is:

$$P(Rec_I|Surv_I) = 1 - P(Ext_{REC}) \approx 1 \tag{25}$$

Concerning the probability of infecting a new resistant hosts the next cropping season ($P(Inf_I|Surv_I, Rec_I)$), the probability for a single propagule with genotype $m_1$ (completely adapted to the resistant host population) to *i*) stay mutant, that is, it does not incur reverse mutations (from mutant $m_1$ to wild type) and *ii*) infect a resistant host is given, respectively, by $(1 - \tau_g)$, where $\tau_g$ is the mutation probability for the infectivity gene $g$, and $e_{max}$, which is the infection probability. Then, the probability for the propagule to mutate and/or not infect any host is $1 - (1 - \tau_g)e_{max}$. Note that we assumed an infinite host population, which allows to neglect the probability to disperse outside the field and the competition with other pathogen genotypes. Two parental infections produce via sexual reproduction a total of $2 \times r_{exp} \times \Gamma_{exp}$ spores, which have $m_1$ genotype with probability $P(Rec_I|m_1) = 0.25$. Consequently, the probability that none of these propagules stays mutant and infects a host in the next cropping season is $P(Ext_{Pr}) = [1 - (1 - \tau_g)e_{max}]^{2 \times r_{exp} \times \Gamma_{exp} \times P(Rec_I)}$. Given the value $\tau_g = 10^{-4}$, $e_{max} = 0.9$, $r_{exp} = 2$ and $\Gamma_{exp} = 14$, $P(Ext_{Pr}) \approx 1 \times 10^{-14}$. Consequently, the probability of infection of new resistant hosts by propagules with genotype $m_1$ is:

$$P(Inf_I|Surv_I, Rec_I) = 1 - P(Ext_{Pr}) \approx 1 \tag{26}$$

The combination of equations (1), (3), (5) and (6) gives:

$$P(Inf_I) = P(Surv_I) \tag{27}$$

Then, the probability of extinction of N infections is as in equation (2).

Above N=50,000 infections, the probability of extinction is less than 1%. Therefore, we chose this threshold to define the time to mutant pathogen establishment.

## Note S5 Insights on competition between superpathogen and single mutants in mixture and mosaic strategies

We analysed the response of two outputs to variation to the cropping ratio for mixture and mosaic strategies, at low mutation probability and for pathogen with clonal reproduction system (Fig. S14). These outputs are: *i*) the time elapsed between the establishment of the two single mutants ($|T_{SM1} - T_{SM_2}|$); *ii*) the proportion of simulations where the superpathogen (SP) gets established before the last SM established. Note that, since the SP never gets established before both the SMs, the proportion of simulations where the SP get established after the two SMs is the complementary proportion of the second output considered. The competition between SP, $SM_1$ and $SM_2$, and thus the relative order of their establishment is a major drive to the U-shaped response of the probability of SP establishment reported in Fig. 3A in the main text. More details are provided in the "Discussion" section in the main text.

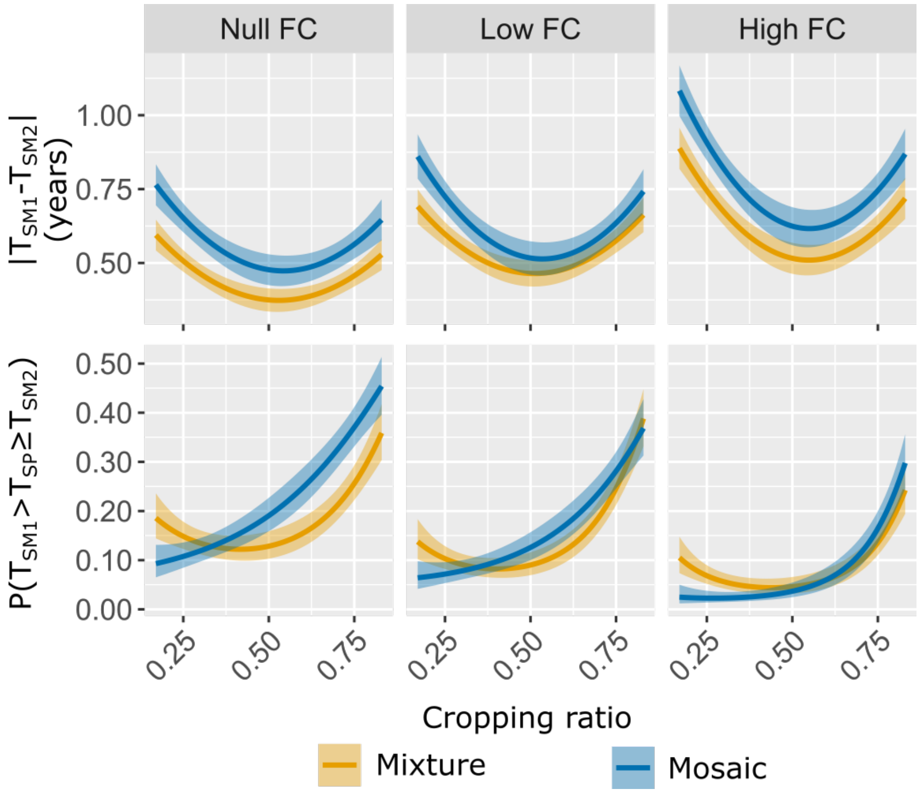


Figure S14: Time elapsed between the establishment of the two single mutants ($|T_{SM1}–T_{SM2}|$) as a function of the cropping ratio for mixture and mosaic strategies (top row). Proportion of simulations where the SP get established before the last SM established as a function of the cropping ratio for mixture and mosaic strategies (bottom row). Curves in the top row (respectively bottom row) are based on the fitting of second-order polynomial (respectively logistic) regression; shaded envelopes delimit the 2.5th and 97.5th percentiles.

# Note S6 Effect of mutation probability on evolutionary outputs

To further investigate the effect of mutation probability on the evolutionary outputs (probability of SP establishment and time to SP establishment), we ran additional simulations considering further mutation probability values ($\tau = \{10^{-7}; 5 \times 10^{-7}; 10^{-6}; 5 \times 10^{-6}; 10^{-5}; 5 \times 10^{-5}; 10^{-4}\}$). In particular, we focused on the pyramiding strategy, where the effect of the mutation probability on the evolutionary outputs is the greatest. Since the results in the main text showed that the type of pathogen reproduction system does not affect the evolutionary outputs for the pyramiding strategy, here, for the sake of simplicity, we only ran simulations for the purely clonal reproduction system. The probability of SP establishment increases from 0 to 1 for increasing value of mutation probability (Fig. S15 A). As a mirror effect, the time to SP establishment decreases with the mutation probability (Fig. S15 B).

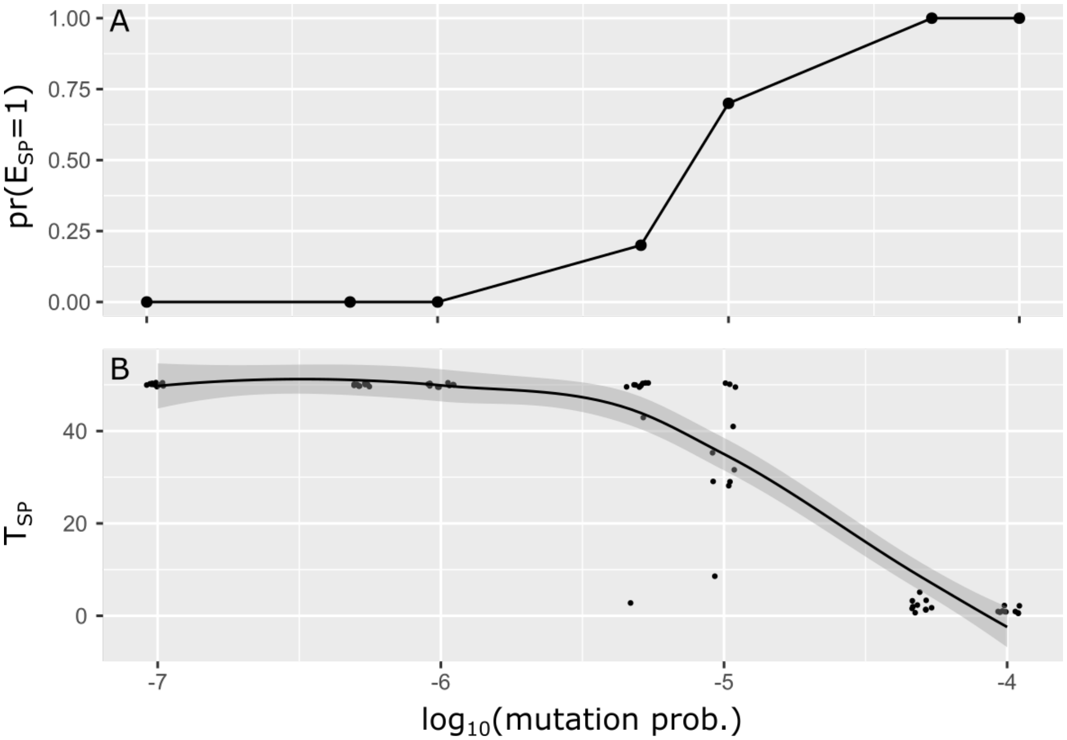


Figure S15: Probability of SP establishment (A) and time to SP establishment (B) for the pyramiding strategy at low fitness cost ($\theta = 0.25$) and intermediate cropping ratio ($\varphi = 0.5$). Panels show the probability of SP establishment and time to SP establishment as a function of the mutation probability. In panel A the curve connects the probabilities of SP establishment computed for the 7 levels of mutation probabilities (10 repetitions each). In panel B, the curve is based on the fitting of local polynomial regression to simulation outputs (represented by points); shaded envelopes delimit the 2.5th and 97.5th percentiles.

# Note S7 Effect of plant-pathogen interaction matrix definition

According to Leonard (1977), the fitness of an adapted pathogen on a resistant host is given by the interplay of: the fitness cost $\theta$ (paid on all hosts) and the advantage $a$ of an adapted pathogen on hosts with corresponding gene of resistance (Table S2). In the literature, several examples of $\{\theta, a\}$ combinations can be found:

1. $\theta = a > 0$, meaning that the adapted pathogen experiences a fitness cost $\theta$ only on the susceptible host, while on the resistant host, $\theta$ is counterbalanced by $a$, *i.e.* the advantage of an adapted pathogen on hosts with corresponding gene of resistance (Fabre et al., 2009, 2015; Djidjou-Demasse et al., 2017; Rimbaud et al., 2018a; Rousseau et al., 2019). In other words, an adapted pathogen only pays a fitness cost for its unnecessary virulences on a given host. For the superpathogen, the fitness costs are multiplicative on the susceptible host.

2. $\theta > 0$ and $a = 0$, meaning that the adapted pathogen experiences the same fitness cost $\theta$ on every host it can infect (Sapoukhina et al., 2009; Lo Iacono et al., 2012; Nilusmas et al., 2020; Watkinson-Powell et al., 2020; Clin et al., 2021, 2022). For the superpathogen, the fitness costs are multiplicative on every host.

3. $\theta = a = 0$, meaning that the adapted pathogen does not experience any fitness cost on any host (Van den Bosch and Gilligan., 2003; Ohtsuki and Sasaki, 2006; Lo Iacono et al., 2013; Wingen et al., 2013; Lof et al., 2017; Lof and van der Werf, 2017; Pacilly et al., 2018, 2019).

The general plant-pathogen interaction matrix representing these three cases is reported in Table S2.

Table S2: Plant-pathogen interaction matrix.

| | | **Host genotype *v*** | | | |
|---|---|---|---|---|---|
| | | **SC** | $\mathbf{RC_1}$ | $\mathbf{RC_2}$ | $\mathbf{RC_{12}}$ |
| **Pathogen genotypes *p*** | **WT** | 1 | 0 | 0 | 0 |
| | $\mathbf{SM_1}$ | 1-$\theta$ | 1-$\theta$ + a | 0 | 0 |
| | $\mathbf{SM_2}$ | 1-$\theta$ | 0 | 1-$\theta$ + $a$ | 0 |
| | **SP** | $(1\text{-}\theta)^2$ | $(1\text{-}\theta)(1\text{-}\theta + a)$ | $(1\text{-}\theta)(1\text{-}\theta + a)$ | $(1\text{-}\theta + a)^2$ |

The matrix gives the coefficient by which the infection probability is multiplied. The value of this coefficient reflects the relative infection probabilities for the wild-type (WT) and adapted (single mutants $SM_1$ and $SM_2$, and superpathogen SP) pathogen genotypes on the susceptible (SC) and resistant cultivars carrying a single major resistance gene (cultivar $RC_1$ and cultivar $RC_2$), or their combination ($RC_{12}$). $\theta$ is the fitness cost of infectivity with respect to the major resistance genes considered and $a$ is the advantage of an adapted pathogen on hosts with corresponding gene of resistance.

In the work presented in the main text, we considered $\theta = a > 0$ (case 1) and $\theta = a = 0$ (case 3). To deepen our analysis, we ran additional simulations considering $\theta > 0$, $a = 0$ (case 2). In particular, we ran 8,000 additional simulations following the simulation plan presented in Section 2.6.2 in the main text. Here, we only considered one landscape structure, consequently, we ran 50 (instead of 250) repetitions for each parameter combination. Firstly, for each combination of resistance deployment strategy, mutation probability, fitness cost and pathogen reproduction system, we fitted second-order polynomial regressions (or second-order logistic regressions) to assess the response of the area under the disease progress curve $AUDPC$ (or SP establishment $E_{SP}$) to variations of cropping ratio. More details on these outputs are presented in section 2.6.3 in the main text. We decided to omit the time before SP establishment output ($T_{SP}$) because, in some cases, the low number of simulations where the SP established does not allow to fit a regression curve. Then, for each parameter combination, we averaged the output values across the 50 repetitions ($E_{SP,case2}$, $AUDPC_{case2}$) and we compared them to the corresponding values obtained for the simulations in the main text ($E_{SP,case1}$, $AUDPC_{case1}$). Consequently, we defined $\Delta pr(E_{SP} = 1) = E_{SP,case2} - E_{SP,case1}$ and $\Delta AUDPC = AUDPC_{SP,case2} - AUDPC_{SP,case1}$. Our aim was to evaluate the effect of the parameter $a$, advantage of an adapted pathogen on hosts with corresponding gene of resistance, on the model outputs ($E_{SP}$ and $AUDPC$).

Firstly, our results indicate that the effect of pathogen reproduction system on evolutionary and epidemiological outputs is the same whether we consider $a = 0$ or $a = \theta$ (Fig. S16-S17). Secondly, considering $a = 0$ rather than $a = \theta$ does not affect the evolutionary and epidemiological outputs for pyramiding at low mutation probability (Fig. S18). Similarly, there is no difference in the probability of $E_{SP}$ for pyramiding at high mutation probability and low fitness cost (Fig. S19). By contrast, at high mutation probability, the $AUDPC$ is smaller for simulations with $a = 0$, with $AUDPC$ reduction up to 60% for high fitness cost and cropping ratio. Finally, for the other strategies the $AUDPC$ is smaller (up to 10% and 25% reduction for, respectively, low and high mutation probabilities) for simulation with $a = 0$. The differences in $AUDPC$ increases with the cropping ratio. The influence of the value of $a$ on the probability of SP establishment is generally minor at low fitness cost. At high fitness cost the establishment of the SP is less frequent when the SP pays the same fitness cost on all the hosts. This is a direct consequence of the globally smaller fitness of mutant pathogens (*i.e.* SM and SP) in a landscape composed of many resistant hosts, as compared with the scenario where they pay a fitness cost only for their unnecessary virulence.

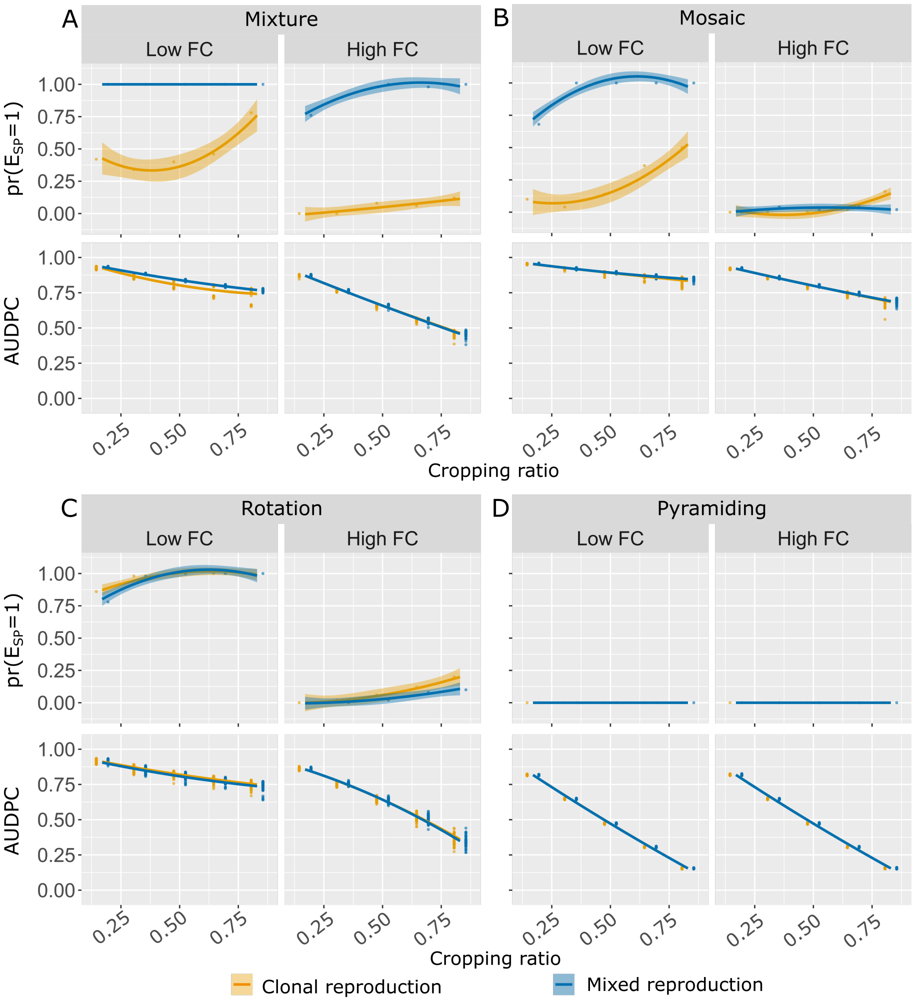


Figure S16: Probability of SP establishment (first row of each panel) and $AUDPC$ (second row) at low ($\tau = 10^{-7}$) mutation probability and at low ($\theta = 0.25$) and high ($\theta = 0.5$) fitness cost (FC). The FC equally reduces the infection rate of an adapted pathogen on all the hosts it infects ($a = 0$). Panels show the probability of $E_{SP}$ and $AUDPC$ as a function of the cropping ratio for the two pathogen reproduction systems and the four deployment strategies considered. Curved are based on logistic or second order polynomial regression fitting performed on simulations outputs (represented by points, note that in the first row of each panel the points represent the proportion of $E_{SP} = 1$ among the 50 replicas), shaded envelopes delimit the 2.5th and 97.5th percentiles.

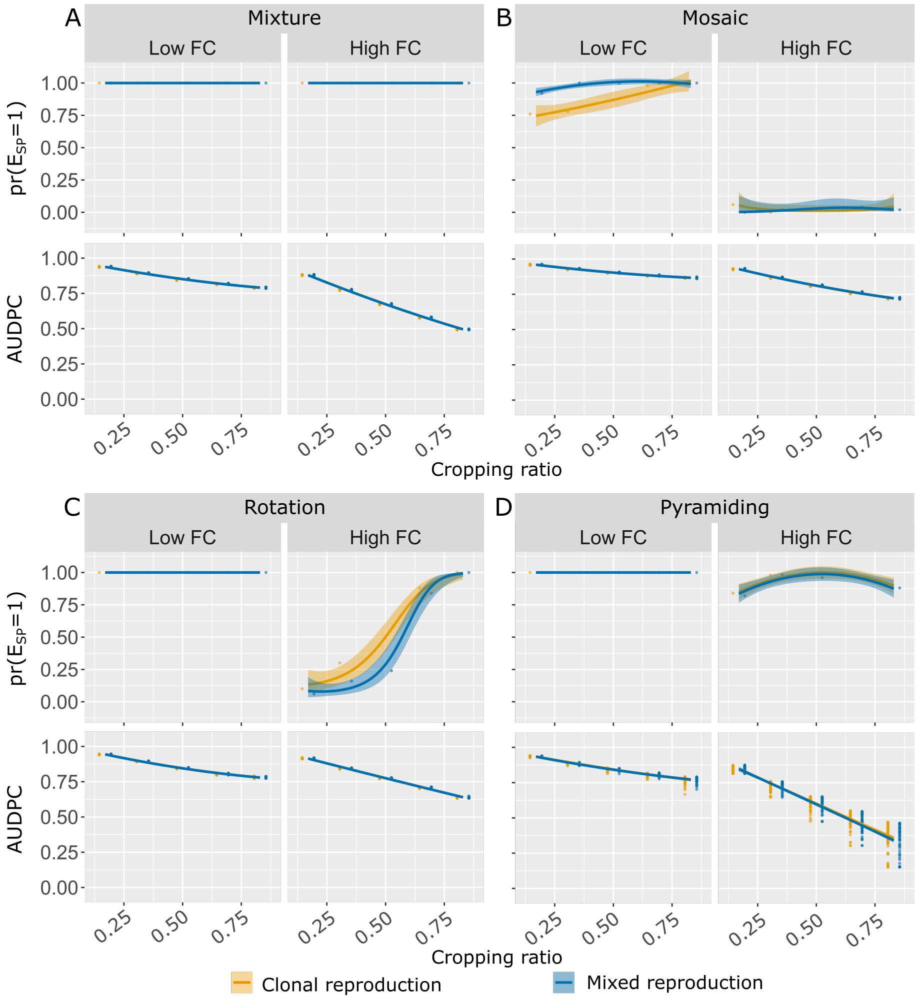


Figure S17: Probability of SP establishment (first row of each panel) and $AUDPC$ (second row) at high ($\tau = 10^{-4}$) mutation probability and at low ($\theta = 0.25$) and high ($\theta = 0.5$) fitness cost (FC). The FC equally reduces the infection rate of an adapted pathogen on all the hosts it infects ($a = 0$). Panels show the probability of $E_{SP}$ and $AUDPC$ as a function of the cropping ratio for the two pathogen reproduction systems and the four deployment strategies considered. Curved are based on logistic or second order polynomial regression fitting performed on simulations outputs (represented by points, note that in the first row of each panel the points represent the proportion of $E_{SP} = 1$ among the 50 replicas), shaded envelopes delimit the 2.5th and 97.5th percentiles.

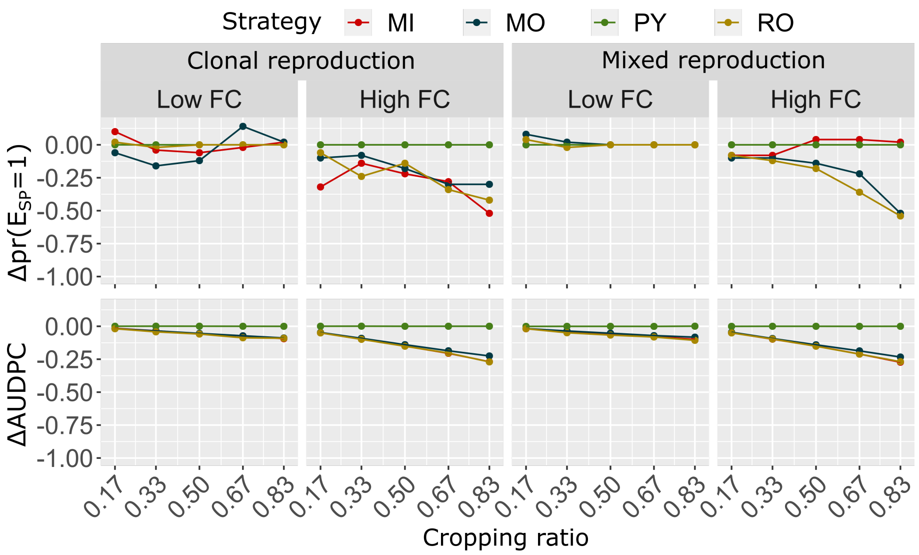


Figure S18: Effect of the type of interaction matrix at low mutation probability ($\tau = 10^{-7}$). Differences in the average probability of SP establishment (first row) and $AUDPC$ (second row) computed between simulations assuming that adapted pathogens 1) only pay a fitness cost for their unnecessary virulences on a given host ($\theta = a > 0$) or 2) pay the same fitness cost on all the host they can infect ($\theta > 0$, $a = 0$). Values of $\Delta pr(E_{SP} = 1)$ and $\Delta AUDPC$ lower than 0 mean, respectively, that the SP get established less frequently and disease severity is lower for $a = 0$ than for $a = \theta$. Panels show the difference in outputs values as a function of the cropping ratio, for the two pathogen reproduction systems, two fitness costs, and the four deployment strategies considered.

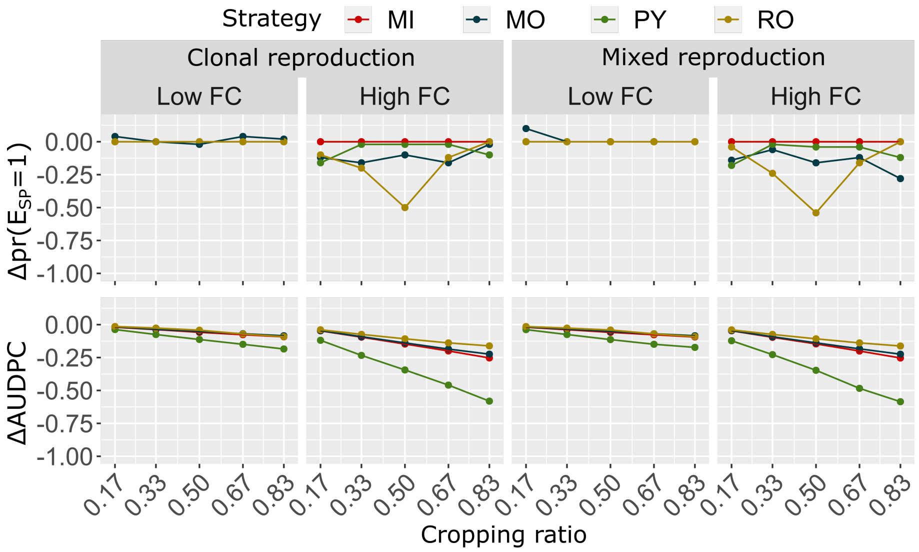


Figure S19: Effect of the type of interaction matrix at high mutation probability ($\tau = 10^{-4}$). Differences in the average probability of SP establishment (first row) and $AUDPC$ (second row) computed between simulations assuming that adapted pathogens 1) only pay a fitness cost for their unnecessary virulences on a given host ($\theta = a > 0$) or 2) pay the same fitness cost on all the host they can infect ($\theta > 0$, $a = 0$). Values of $\Delta pr(E_{SP} = 1)$ and $\Delta AUDPC$ lower than 0 mean, respectively, that the SP get established less frequently and disease severity is lower for $a = 0$ than for $a = \theta$. Panels show the difference in outputs values as a function of the cropping ratio, for the two pathogen reproduction systems, two fitness costs, and the four deployment strategies considered.

# References


Anderson, R. M. and May, R. M. (1992). *Infectious diseases of humans: dynamics and control*. Oxford university press.

Angelotti, F., Hamada, E., Magalhães, E. E., Ghini, R., Garrido, L., and Pedro, M. J. (2017). Climate change and the occurrence of downy mildew in Brazilian grapevines. *Pesquisa Agropecuaria Brasileira*, 52(6):426–434.

Boso, S. and Kassemeyer, H. H. (2008). Different susceptibility of European grapevine cultivars for downy mildew. *Vitis*, 47(1):39–49.

Bouvier, A., Kiêu, K., Adamczyk, K., and Monod, H. (2009). Computation of the integrated flow of particles between polygons. *Environmental Modelling & Software*, 24(7):843–849.

Bove, F., Bavaresco, L., Caffi, T., and Rossi, V. (2019). Assessment of Resistance Components for Improved Phenotyping of Grapevine Varieties Resistant to Downy Mildew. *Frontiers in Plant Science*, 10(November):1–10.

Bove, F., Savary, S., Willocquet, L., and Rossi, V. (2020). Simulation of potential epidemics of downy mildew of grapevine in different scenarios of disease conduciveness. *European Journal of Plant Pathology*, 158:599–614.

Caffi, T., Gilardi, G., Monchiero, M., and Rossi, V. (2013). Production and release of asexual sporangia in Plasmopara viticola. *Phytopathology*, 103(1):64–73.

Caffi, T., Legler, S. E., González-Domínguez, E., and Rossi, V. (2016). Effect of temperature and wetness duration on infection by Plasmopara viticola and on post-inoculation efficacy of copper. *European Journal of Plant Pathology*, 144(4):737–750.

Clark, J. S. C. and Spencer-Phillips, P. T. N. (2004). *Advances in Downy Mildew Research*, volume 2.

Clin, P., Grognard, F., Mailleret, L., Val, F., Andrivon, D., and Hamelin, F. M. (2021). Taking advantage of pathogen diversity and immune priming to minimize disease prevalence in host mixtures: a model. *Phytopathology®*, 111(7):1219–1227.

Clin, P., Grognard, F., Andrivon, D., Mailleret, L. & Hamelin, F. (2022) Host mixtures for plant disease control: Benefits from pathogen selection and immune priming. *Evolutionary Applications*. **15**, 967-975

Delmas, E. L., Fabre, F., Jolivet, J., Mazet, I. D., Richart Cervera, S., Delière, L., and Delmotte, F. (2016). Adaptation of a plant pathogen to partial host resistance : selection for greater aggressiveness in grapevine downy mildew. *Evolutionary Applications*.

Delmas, E. L., Fabre, F., Jolivet, J., Mazet, I. D., Richart Cervera, S., Delière, L., and Delmotte, F. (2016). Adaptation of a plant pathogen to partial host resistance : selection for greater aggressiveness in grapevine downy mildew. *Evolutionary Applications*.

Djidjou-Demasse, R., Moury, B., and Fabre, F. (2017). Mosaics often outperform pyramids: Insights from a model comparing strategies for the deployment of plant resistance genes against viruses in agricultural landscapes. *New Phytologist*.

Fabre, F., Bruchou, C., Palloix, A. & Moury, B. (2009) Key determinants of resistance durability to plant viruses: Insights from a model linking within- and between-host dynamics. *Virus Research*.

Fabre, F., Rousseau, E., Mailleret, L. & Moury, B. (2015) Epidemiological and evolutionary management of plant resistance: optimizing the deployment of cultivar mixtures in time and space in agricultural landscapes. *Evolutionary Applications*.

Frantzen, J. and Van den Bosch, F. (2000). Spread of organisms: can travelling and dispersive waves be distinguished? *Basic and Applied Ecology*, 1(1):83–92.

Grosdidier, M., Ioos, R., Husson, C., Cael, O., Scordia, T., and Marçais, B. (2018). Tracking the invasion: dispersal of hymenoscyphus fraxineus airborne inoculum at different scales. *FEMS microbiology ecology*, 94(5):fiy049.

Kennelly, M. M., Gadoury, D. M., Wilcox, W. F., Magarey, P. A., and Seem, R. C. (2005). Seasonal development of ontogenic resistance to downy mildew in grape berries and rachises. *Phytopathology*, 95(12):1445–1452.

Kennelly, M. M., Gadoury, D. M., Wilcox, W. F., Magarey, P. A., and Seem, R. C. (2007). Primary infection, lesion productivity, and survival of sporangia in the grapevine downy mildew pathogen Plasmopara viticola. *Phytopathology*, 97(4):512–522.

Lalancette, N. (1988). A Quantitative Model for Describing the Sporulation of Plasmopara viticola on Grape Leaves. *Phytopathology*, 78(10):1316.

Lalancette, N., Ellis, M. A., and Madden, L. V. (1988). Development of an Infection Efficiency Model for Plasmopara viticola on American Grape Based on Temperature and Duration of Leaf Wetness.

Leonard, K. (1977). Selection pressures and plant pathogens. *Annals of the New York Academy of Sciences*, 287(1):207–222.

Lof, M. E., Vallavieille-Pope, C. & van der Werf, W. (2017a). Achieving durable resistance against plant diseases: scenario analyses with a national-scale spatially explicit model for a wind-dispersed plant pathogen. *Phytopathology*. **107**, 580-589

Lof, M. E. and van der Werf, W. (2017b). Modelling the effect of gene deployment strategies on durability of plant resistance under selection. *Crop Protection*, 97:10–17.

Lo Iacono, G., van den Bosch, F., and Paveley, N. (2012). The evolution of plant pathogens in response to host resistance: Factors affecting the gain from deployment of qualitative and quantitative resistance. *Journal of Theoretical Biology*, 304:152–163.

Lo Iacono, G., Van den Bosch, F. & Gilligan, C. (2013) Durable resistance to crop pathogens: an epidemiological framework to predict risk under uncertainty. *PLoS Computational Biology.* **9**, e1002870

Mouafo-Tchinda, R. A., Beaulieu, C., Fall, M. L., and Carisse, O. (2020). Effect of temperature on aggressiveness of Plasmopara viticola f. sp. aestivalis and P. viticola f. sp. riparia from eastern Canada. *Canadian Journal of Plant Pathology*, 43(1):73–87.

Mundt, C. C., Sackett, K. E., Wallace, L. D., Cowger, C., and Dudley, J. P. (2009). Long-distance dispersal and accelerating waves of disease: Empirical relationships. *American Naturalist*, 173(4):456–466.

Nilusmas, S., Mercat, M., Perrot, T., Djian-Caporalino, C., Castagnone-Sereno, P., Touzeau, S., Calcagno, V. & Mailleret, L. (2020) Multi-seasonal modelling of plant-nematode interactions reveals efficient plant resistance deployment strategies. *Evolutionary Applications.* **13**, 2206-2221

Ohtsuki, A. & Sasaki, A. (2006) Epidemiology and disease-control under gene-for-gene plant–pathogen interaction. *Journal Of Theoretical Biology.* **238**, 780-794

Ojiambo, P. S., Gent, D. H., Mehra, L. K., Christie, D., and Magarey, R. (2017). Focus expansion and stability of the spread parameter estimate of the power law model for dispersal gradients. *PeerJ*, 2017(6):1–20.

Pacilly, F., Hofstede, G., Bueren, E., Kessel, G. & Groot, J. (2018). Simulating crop-disease interactions in agricultural landscapes to analyse the effectiveness of host resistance in disease control: The case of potato late blight. *Ecological Modelling.* **378** pp. 1-12

Pacilly, F., Hofstede, G., Bueren, E. & Groot, J. (2019) Analysing social-ecological interactions in disease control: an agent-based model on farmers' decision making and potato late blight dynamics. *Environmental Modelling & Software.* **119** pp. 354-373

Papaïx, J., Adamczyk-Chauvat, K., Bouvier, A., Kiêu, K., Touzeau, S., Lannou, C., and Monod, H. (2014a). Pathogen population dynamics in agricultural landscapes: The Ddal modelling framework. *Infection, Genetics and Evolution*, 27:509–520.

Papaïx, J., Burdon, J. J., Zhan, J., and Thrall, P. H. (2015). Crop pathogen emergence and evolution in agro-ecological landscapes. *Evolutionary Applications*, 8(4):385–402.

Papaïx, J., Touzeau, S., Monod, H., and Lannou, C. (2014b). Can epidemic control be achieved by altering landscape connectivity in agricultural systems? *Ecological Modelling*, 284:35–47.

Rimbaud, L., Papaïx, J., Rey, J.-F., Barrett, L. G., and Thrall, P. H. (2018). Assessing the durability and efficiency of landscape-based strategies to deploy plant resistance to pathogens. *PLoS computational biology*, 14(4):e1006067.

Rimbaud, L., Papaïx, J., Barrett, L. G., Burdon, J. J., and Thrall, P. H. (2018a). Mosaics, mixtures, rotations or pyramiding: What is the optimal strategy to deploy major gene resistance? *Evolutionary Applications*, 11(10):1791–1810.

Rossi, V., Giosuè, S., and Caffi, T. (2009). Modelling the dynamics of infections caused by sexual and asexual spores during Plasmopara Viticola epidemics. *Journal of Plant Pathology*, 91(3):615–627.

Rousseau, E., Bonneault, M., Fabre, F., Moury, B., Mailleret, L. & Grognard, F. (2019) Virus epidemics, plant-controlled population bottlenecks and the durability of plant resistance. *Philosophical Transactions Of The Royal Society B.*

Rumbolz, J., Wirtz, S., Kassemeyer, H. H., Guggenheim, R., Schäfer, E., and Büche, C. (2002). Sporulation of Plasmopara viticola: Differentiation and light regulation. *Plant Biology*, 4(3):413–422.

Sapoukhina, N., Durel, C. E., and Le Cam, B. (2009). Spatial deployment of gene-for-gene resistance governs evolution and spread of pathogen populations. *Theoretical Ecology*, 2:229–238.

Van den Bosch, F. & Gilligan, C. (2003) Measures of durability of resistance. *Phytopathology.* **93**, 616-625

Watkinson-Powell, B., Gilligan, C. A., and Cunniffe, N. J. (2020). When does spatial diversification usefully maximize the durability of crop disease resistance? *Phytopathology*, 110(11):1808–1820.

Wingen, L., Shaw, M. & Brown, J. (2013) Long-distance dispersal and its influence on adaptation to host resistance in a heterogeneous landscape. *Plant Pathology.* **62**, 9-20
